# The Experience of Healthy Pregnancy in High Parity Women: A Phenomenological Study in North Jordan

**DOI:** 10.3390/medicina57080853

**Published:** 2021-08-22

**Authors:** Ghadeer Alzboon, Gülşen Vural

**Affiliations:** 1 Department of Nursing Sciences, Irbid National University, Irbid 21110, Jordan; 2 Department of Birth and Women’s Health Nursing, Near East University, Nicosia 99138, North Cyprus, Turkey; gulsenural@hotmail.com

**Keywords:** experience, Jordan, parity, pregnancy, quality of life

## Abstract

*Background and Objectives*: High parity women are more likely to have poor quality of life during pregnancy than low parity women. Thus, the aim of this study was to explore the lived experience of healthy pregnancy among high parity women in North Jordan. *Materials and Methods*: A descriptive phenomenological design was employed in this study to complement previously published quantitative results. Fourteen pregnant women, who had four children or more, were recruited purposely according to inclusion criteria from Irbid city in North Jordan. Data were collected using face-to-face, semi-structured interviews. Colaizzi’s method was employed to analyze the verbatim data. *Results*: There were three main themes which emerged from participants significant statements: they had new discomforts, antenatal care and follow-up, and social issues. Each extracted theme was linked to some factors (subthemes), which had a positive or negative impact on the quality of life of high parity women during pregnancy. High parity women who experienced multiple stressors had a poor quality of life. *Conclusions*: Experiencing new discomforts, less or no antenatal care, and a lack of social support negatively affected the quality of life among high parity women. Antenatal interventions should be designed based on high parity women’s perceptions of their health and wellbeing in order to improve their quality of life and ultimately prevent maternal morbidity and mortality. Further quantitative studies are needed to explore the impact of previous mentioned factors on maternal quality of life and outcomes.

## 1. Introduction

Pregnancy is generally viewed as a normal experience in a woman’s life. Nevertheless, many studies have documented that women’s quality of life (QOL) decreases during pregnancy [1,2]. QOL is multi-dimensional concept encompassing physical, psychological, and social health [3,4]. Parity is one factor that may be associated with women’s health and QOL; when describing the impact of this factor, the associated literature often uses the terms “primiparous” versus “multiparous” or “low parity” versus “high parity” refer to women who have given birth once versus those who have done so multiple times.

High parity women are more likely to have adverse maternal and neonatal outcomes, such as antenatal depression, cesarean section, and neonatal infant mortality [5,6,7]. Moreover, high parity women have been shown to be less satisfied with life and to have a poorer QOL when compared with low parity women [3,8,9,10]. A mixed method design was employed with quantitative and qualitative method of inquiry. The quantitative part has been previously published and showed that women who had four children or more (i.e., who were categorized as high parity) had lower QOL scores than pregnant women who did not have children yet or who had only one child [11].

However, the measuring tools have not fully illustrated why high parity women have a poorer QOL, and the factors that have been investigated are limited. There is a lack of knowledge regarding the experience of high parity women during pregnancy. Therefore, the qualitative research was carried out to complement the quantitative findings, and aimed to describe and understand the experience of healthy pregnancy among high parity women (i.e., those who have four children or more) in Jordan. The focus of the findings was the scope of the women’s QOL and its influencing factors.

The findings can inform maternal health professionals about the needs and concerns of high parity women in pregnancy. They can also be useful to those designing educational and counseling interventions during antenatal care. Consequently, the QOL and health of high parity women could be improved during pregnancy. To achieve these ends, the primary research question explored in this study was as follows: What is the lived experience of pregnancy among high parity women (who are considered healthy) in Irbid, Jordan?

## 2. Materials and Methods

### 2.1. Study Design

A descriptive phenomenological design was utilized in this study. This is an attractive qualitative approach to inquiry because it generates rich new descriptions about phenomena from the perspectives of those who experience them firsthand [12]. In this study, the experiences of healthy pregnancy among women with four or more children were described from their own perspectives.

### 2.2. Study Setting

Participants were identified by first contacting the attendees (including women of different ages and who were not pregnant) of two fitness centers in one area of Irbid, Jordan. Generally, the Jordanian people who live in this region are socially active and know each other. Thus, they were able to direct the researchers to pregnant women with four or more children. The interviews were carried out in a place that was convenient for the participants.

### 2.3. Sample

#### 2.3.1. Sampling Technique

The participants were selected in three steps. First, the researchers met the attendees (who were women) of two fitness centers in an area close to the researchers. They were asked if they knew any pregnant women who had four or more children. In this step, 19 pregnant women who had 4 or more children were reached. Second, a snowball sampling technique was used to select other high parity pregnant women. Specifically, the potential participants (19 pregnant women) who were accessed in the first step were asked to find other participants. In this step, nine additional pregnant women were reached. Third, a purposive sampling technique was employed to determine the eligibility of the participants. As such, the 19 and 9 pregnant women identified in the first and second steps, respectively, were contacted to determine their eligibility.

#### 2.3.2. Sample Size and Inclusion Criteria

The sample size is relatively small in qualitative research. Creswell suggested using a sample size of a minimum of 3 to a maximum of 15 participants in a phenomenological study [13]. Therefore, in this study, the number of participants was 14.

The participants who were included in this study were Jordanian in nationality, were married, were able to read and write in the Arabic language, had four children or more, were pregnant with a single fetus, and had conceived naturally. The exclusion criteria were as follows: being a smoker, having had any previous or current obstetric health problems (e.g., miscarriage or gestational diabetes), and having had any previous or current systematic health problems (e.g., diabetes mellitus or hypertension).

### 2.4. Data Collection

Data were collected during a period of about one month using face-to-face, semi-structured interviews that were recorded with a digital voice recorder. The participants were given a written information sheet about the study before they consented to participate. The information sheet included the following items: the purpose of the research, the risk and benefit of the study, information about voluntary participation and withdrawing from the study, the contact information of the researchers, and the date of the interview.

For each participant, the first interview began with questions about their socio-demographic data (i.e., age, income, educational level, and other characteristics) and obstetric data (i.e., gestational age, number of antenatal visits, pregnancy planning, and other characteristics).

In first interview, the researchers asked the primary research question (“What is your lived experience of pregnancy while you have four children or more?”), and this was followed up with other open-ended questions to get more information about the topic of conversation. Some examples of these follow-up questions are, “Can you give an example of that?”, and “What did you think about that?”. On average, each participant was interviewed twice; the interviews ceased when repetitive statements began to be made by the participant. Each interview lasted around 30–45 min. The quality of the interview and the transcribed data were checked by a nurse who is an expert in qualitative research. However, the primary investigator was qualified by education and training on qualitative research.

### 2.5. Data Analysis and Evaluation

The transcribed interviews were analyzed using Colaizzi’s method [14], which includes seven steps. (1) Read all participants’ descriptions of their experiences after transcribing the recorded interviews. (2) Extract significant statements. (3) Formulate meanings from the significant statements. (4) Conceptualize the aggregate formulated meanings into themes. (5) Categorize the obtained concepts. (6) Construct comprehensive descriptions of the phenomenon under study. (7) Validate the findings using Lincoln and Cuba’s method of evaluation [15]. As such, the significant statements of each participant were documented, categorized, and anonymously labeled e.g., participant 1 was labeled “P1” to adhere to the required ethical research standards.

The trustworthiness of data were assessed using Lincoln and Guba’s method of evaluation [15]. This method consists of four measures: credibility, dependability, confirmability, and transferability. The credibility check was carried out by returning the transcribed descriptions to the participants themselves to have them check their truth, and by comparing the transcribed interviews of the participants at different points in time. Furthermore, the research process was reviewed by another nursing qualitative researcher to improve its dependability. To ensure the confirmability of the findings, the researchers documented all the steps of the study, including data collection and data analysis. Additionally, a heterogeneous sample was selected that included participants with varied socio-demographic and obstetric characteristics, to achieve transferability.

### 2.6. Ethical Considerations

Ethical approval was obtained from the Institutional Review Board of Near East University (Code Number: YDU/2020/78-984). Written and verbal informed consent was obtained from all pregnant women to participate voluntary in this study and to publish this paper. All the data were labeled with non-descriptive identifiers. These codes were used instead of the participants’ names and addresses.

## 3. Results

The characteristics of the participants were described in Table 1. The total number of participants in this study was 14. Half of them were over 35 year and were housewives. The majority of them (42.8%) had monthly income of 450–800 Jordan Dinar. The number of participants in each trimester of pregnancy relatively was equal. Half of participants planned their pregnancy, and 71.4% of participants sought antenatal care (see Table 1).

As summarized in Table 2, the interview results were reported as extracted themes and subthemes from the verbatim transcriptions. The main themes were: having new discomforts, antenatal care and follow-up, and social issues.

### 3.1. Theme 1: Having New Discomforts

In this study, the participants stated that they were experiencing new types of discomfort in their current pregnancy. Their perceptions were categorized into two subthemes: advanced maternal age and having additional responsibilities. The participants with advanced maternal age were categorized as those older than 35. In this study, the mean age of the participants was 35. Some participants thought that experiencing new types of discomfort, which were not present in previous pregnancies, was related to their current age and having additional duties, such as having additional physical work and being caregiver to one family member (see Table 2). Generally, most participants carried out routine daily duties such as cooking and child rearing.

### 3.2. Theme 2: Antenatal Care and Follow Up

The transcribed statements from the interviews related to the second theme revealed three subthemes: perceptions after having previous experiences of pregnancy, baby gender preferences, and unplanned/unwanted pregnancy. High parity women perceived themselves as having good knowledge from their previous experiences of pregnancy, so they did not need to seek antenatal care. Another participant’s quotes revealed that participants whose baby was not their preferred gender tended not to seek antenatal care. All participants with planned pregnancy had either one boy or girl. Most of them made only one antenatal visit to check the baby gender. Their preference was only to achieve family balance. Furthermore, participants who were experiencing an unplanned and unwanted pregnancy sought no antenatal checkup or care.

### 3.3. Theme 3: Social Issues

This theme was found to be composed of three subthemes: low socioeconomic status, social support, and the role of other children’s ages. The findings showed that four participants had low incomes and complained of financial hardship. The participants’ descriptions revealed the impact of a low income in women’s general health. Concerning social support, most women received emotional support from their husband, mother, or sisters, with some variations between participants. However, in general, the instrumental support they received was limited. Regarding the role of other children’s ages, most high parity pregnant women engaged in birth spacing, so their eldest children tended to be older than 13. The extracted statements revealed that older children provided instrumental social support to the pregnant women.

## 4. Discussion

The purpose of this qualitative study was to explore and discover the lived experiences of pregnancy among high parity women (i.e., those who have four children or more). Specifically, the focus of the study was to explore high parity women’s health and QOL during pregnancy, and the related factors. The results showed three main themes: having new discomforts, antenatal care and follow-up, and social issues.

### 4.1. Having New Discomforts

During the interviews, most of the women complained of new discomforts that they had not experienced in previous pregnancies. They attributed these discomforts to having an advanced age and having new duties, which contributed to the development of both discomforts and the deterioration of their health. It has been hypothesized that aging process caused changes in the maternal cardiovascular, immune, and endocrine systems [16]. This was supported by the findings of one systematic review and meta-analysis [17], which found an association between advanced maternal age and maternal complications during pregnancy, such as preeclampsia and gestational diabetes.

Furthermore, some pregnancy-related discomforts are experienced primarily by women who have high parity and an advanced maternal age [18,19]. In one study, urinary incontinence was found to be most common among high parity women and to have a negative effect on women’s QOL [20]. It was associated with poor work performance, being less likely to perform daily home activities, and increased anxiety [18]. Further research is required to investigate pregnancy-related discomforts and their effects on high parity women’s health and QOL.

Additionally, women generally have a number of social roles, such as wife, daughter, employee, and mother [21]. Pregnancy is also considered a natural role. As such, pregnancy adds new responsibilities to the daily routines of women, which may contribute to adverse effects on their mental and physical health [21,22] and lead to a poor QOL. A Brazilian study found that high parity women, who have two or more children, may be unable to cope effectively with the parenting stress that accompanies having a new baby in addition to that associated with caring for their other children, increasing their risk for antenatal depression [23]. An assessment of the stress factors and available coping resources of high parity women is required in order to promote their physiological and psychological health during pregnancy. Further research is recommended to investigate the pregnancy related symptoms in relation to the QOL of high parity women.

### 4.2. Antenatal Care and Follow Up

High parity women have been found to use antenatal health services less frequently than low parity women do [24,25]. The results of the current study were in line with this, and three subthemes were identified that influence pregnant women’s engagement in antenatal care: perceptions after having previous experiences of pregnancy, baby gender preferences, and unplanned or unwanted pregnancy.

In one study [26], it was found that high parity women who had a positive previous experience of pregnancy felt comfortable and relaxed, and that they needed little or no antenatal care. They believed that antenatal care was important and necessary for primiparous women because they had more to learn [26]. This was consistent with findings of this study.

Regarding the gender of the baby, a qualitative study in Iran found that the gender of the fetus was one a pregnancy-related concern that could affect women’s QOL during pregnancy [4]. Pregnant women with a female fetus were more likely to receive inadequate antenatal care than women who had male fetus [27]. In this study, participants did not differentiate between boy and girl but they preferred to get varied gender in order to achieve family balancing.

It was further found that women who had an unwanted pregnancy postponed their antenatal care and attended fewer antenatal visits [28]. Unplanned pregnancy has been linked to a poor QOL, less antenatal care, and risky behaviors [29]. Less or inadequate antenatal care has been associated with adverse perinatal outcomes, such as stillbirth and congenital malformation [30]. Generally, antenatal care is a primary intervention that contributes in decreasing maternal morbidity and mortality [31]. In previous evidence, Antenatal care allows to reveal women’s self-reported wellbeing and identifying the health-related burdens of pregnancy. Consequently, proper and recommended interventions can be addressed and provided for women, particularly those with high parity, to increase their utilization of antenatal health services which promote women’s health and QOL.

### 4.3. Social Issues

The transcribed statements related to this theme could be categorized into three subthemes: low socioeconomic status, social support, and the role of other children’s ages. One study reported a correlation between poor sleep quality and low socioeconomic status. Silva-Perez et al. found that low income, poor QOL, and poor diet increased the risk of adverse maternal and neonatal outcomes [32].

The findings of the current study showed that most pregnant women seek emotional and instrumental support from their family members, such as their husband, children, and other family members. Many studies have found that pregnant women receive high levels of social support and are satisfied with the support they receive from their husband, family, and friends [11,27,33].

Concerning the role of other children’s ages, it was found that the women’s older children helped them by performing some of the housework duties. Thus, older children can be considered a source of support for the family. Receiving more social support could promote women’s physical, psychological, and social health, and lead to a better QOL during pregnancy [34]. Social support interventions should be included in maternal health promotion programs, particularly for high parity women.

The findings of this study have revealed that there are both positive and negative factors that influence high parity women’s health and QOL during pregnancy. Specifically, having a high socioeconomic status and a planned pregnancy may be considered positive factors, while having additional and new duties to perform may contribute to illness and a poor QOL. In summary, based on the interviews in this study, high parity women were found to have a unique experience during pregnancy. They may experience multiple stressors and protective factors simultaneously. Thus, there is no single, independent factor linked to high parity women’s health or QOL. Pregnant women with multiple stressors experience negative maternal health consequences, such as hyperemesis gravidarum and preterm birth [35]. However, providing proper and early interventions will advance the quality of the antenatal care women receive, improve high parity women’s QOL, and, therefore, decrease maternal and neonatal morbidity and mortality.

### 4.4. Limitations

The study has two limitations: the use of a small sample size and the possibility of selection bias. However, the qualitative method of inquiry that was employed here typically relies on a small sample size, because the aim is not to generate generalizable findings, as is the case for the quantitative method. Regarding selection bias, it is possible that the participants omitted to tell the researchers about previous obstetric or medical health problems, which would result in inconsistency with the inclusion criteria of the study. In future research, we recommend recruiting a sample from maternal healthcare settings, as using participant medical files could confirm their eligibility.

## 5. Conclusions

The narrative descriptions of high parity women revealed three main issues: having new discomforts, antenatal care and follow-up, and social issues. In relation to each of these themes, it was found that high parity women experience both positive and negative factors that can affect their health and QOL during pregnancy. Some factors were indirectly affect the QOL of high-parity pregnant women such as advance maternal age, unwanted pregnancy, and low income. Moreover, exposure to multiple stressors simultaneously during pregnancy could adversely affect women’s physical, psychological, social wellbeing and ultimately poor QOL.

As such, healthcare providers should assess pregnant women’s history of pregnancy and perform a complete physical assessment of common discomforts experienced during pregnancy. In doing so, healthcare providers can also direct women to appropriate sources of social support. High parity women should be empowered to seek out antenatal care through increased awareness of its importance. Further research is required to investigate quantitatively the role of the themes and subthemes identified in this study, which are considered factors influencing high parity women’s QOL and maternal and neonatal health outcomes.

## Figures and Tables

**Table 1:** Characteristics of participants (*n* = 14).

Variables	Frequency	%
Age		
less than 29 years old	1	7.2
30–34	6	42.8
35–40	7	50.0
Educational level		
Primary school	2	14.3
Secondary school	5	35.7
College diploma	2	14.3
Bachelor’s degree	3	21.4
Graduate degree	2	14.3
Occupation		
Housewife	7	50.0
Part-time worker	1	7.2
Full-time worker	6	42.8
Total monthly family income		
Less than JD * 450	4	28.6
JD450–800	6	42.8
More than JD 800	4	28.6
Parity		
4	11	78.6
5 or more	3	21.4
Gestational age		
1st trimester	4	28.6
2nd trimester	5	35.7
3rd trimester	5	35.7
Planned pregnancy		
Yes	7	50.0
No (wanted)	3	21.4
No (unwanted)	4	28.6
Antenatal care		
Yes	10	71.4
No	4	28.6
Total	14	100.0

* Jordan dinar, JD 1 = USD 1.41.

**Table 2:** Summary of emergent themes and subthemes of participants’ descriptions.

Themes	Subthemes	Supporting Quotes
1. Having new discomforts	Advanced maternal age	“I feel severe back pain. when pregnant women become older and older, the pregnancy become more difficult. I feel very tired and exhausted in this pregnancy, not as in previous pregnancies” (P10) *.
	Impact of new and additional duties	“I have varicose veins in my legs and in the genital area that did not appear in previous pregnancies; I feel difficulty when I want to sit down. I think that was related to working (picking olives), which takes a lot of physical effort” (P4).
2. Antenatal care and follow up	Perceptions after having previous experiences of pregnancy	“I have borne 4 children and this number 5; I know what pregnant women should do. no need to visit an obstetrician or antenatal health center” (P7).
	Baby gender preferences	“I want a brother to my son, but….Oh (her tone of voice and facial expressions reflect her unhappiness toward the gender of her baby) no need for an antenatal checkup” (P1).
	Unplanned/unwanted pregnancy	“I do not want this pregnancy. I do not take vitamins or go to the doctor for antenatal checkups” (P12).
3. Social issues	Low socioeconomic status	“We have a new home under construction (they lived in an old, rented two-bedroom house with poor ventilation), and there are a lot of things to do. We did not have the money to bring workers into clean the area after the construction workers left, so I did that with my husband” (P5).
	Social support	“I did many things at home. My husband does not participate in housework or child care. But he listens to me, solves any problem that happen with us” (P2). “I feel comfort when I talk with my sister or my mother or visit them” (P6).
	The role of other children’s ages	“I have many roles and responsibilities, but my older daughter (14 years old) helps me in doing some home duties” (P9).

* Pn: Participant (number); each participant was labeled using numbers (i.e., P1, P2, etc.).

## Data Availability

This research article was part of doctoral thesis entitled “The quality of life and its influencing factors of healthy pregnant women in north of Jordan” which is available online at: http://docs.neu.edu.tr/library/6916287653.pdf (accessed on 25 June 2020).
